# UCP2 -866G/A, Ala55Val and UCP3 -55C/T Polymorphisms in Association with Obesity Susceptibility — A Meta-Analysis Study

**DOI:** 10.1371/journal.pone.0058939

**Published:** 2013-04-01

**Authors:** Li Qian, Kuanfeng Xu, Xinyu Xu, Rong Gu, Xuan Liu, Shan Shan, Tao Yang

**Affiliations:** Department of Endocrinology, The First Affiliated Hospital of Nanjing Medical University, Nanjing, Jiangsu, China; Sapienza University, Italy

## Abstract

**Aims/hypothesis:**

Variants of UCP2 and UCP3 genes have been reported to be associated with obesity, but the available data on the relationship are inconsistent. A meta-analysis was performed to determine whether there are any associations between the UCP2 -866G/A, Ala55Val, and UCP3 -55C/T polymorphisms and obesity susceptibility.

**Methods:**

The PubMed, Embase, Web of Science and CNKI, CBMdisc databases were searched for all relevant case-control studies. The fixed or random effect pooled measure was determined on the bias of heterogeneity test among studies. Publication bias was examined by the modified Begg's and Egger's test.

**Results:**

Twenty-two published articles with thirty-two outcomes were included in the meta-analysis: 12 studies with a total of 7,390 cases and 9,860 controls were analyzed for UCP2 -866G/A polymorphism with obesity, 9 studies with 1,483 cases and 2,067 controls for UCP2 Ala55Val and 8 studies with 2,180 cases and 2,514 controls for UCP3 -55C/T polymorphism. Using an additive model, the UCP2 -866G/A polymorphism showed no significant association with obesity risk in Asians (REM OR = 0.81, 95% CI: 0.65–1.01). In contrast, a statistically significant association was observed in subjects of European descent (FEM OR = 1.06, 95% CI: 1.01–1.12). But neither the UCP2 Ala55Val nor the UCP3 -55C/T polymorphism showed any significant association with obesity risk in either subjects of Asian (REM OR = 0.84, 95% CI: 0.67–1.06 for Ala55Val; REM OR = 0.94, 95% CI: 0.55–1.28 for -55C/T) or of European descent (REM OR = 1.04, 95% CI: 0.80-1.36 for Ala55Val; FEM OR = 1.08, 95% CI: 0.97–1.20 for -55C/T).

**Conclusions and Interpretation:**

Our meta-analysis revealed that the UCP2 -866G/A polymorphism may be a risk factor for susceptibility to obesity in subjects of European descent, but not in individuals of Asian descent. And our results did not support the association between UCP2 Ala55Val, UCP3 -55C/T polymorphisms and obesity in the populations investigated. This conclusion warrants confirmation by further studies.

## Introduction

Uncoupling proteins, which comprise five UCP homologues (UCP1-UCP5), belong to the family of mitochondrial transporter proteins that uncouple oxidative phosphorylation from ATP synthesis and release excess energy as heat [1]. Among the five UCP homologues, UCP2 and UCP3 genes are situated close to each other on chromosome 11q13. UCP2 is widely distributed in all tissue types, with predominant expression in white adipose tissue and skeletal muscle [2], whereas UCP3 expression is mostly restricted to skeletal muscle [3]. Although the physiological roles of UCP2 and UCP3 are less well established, most studies suggested that UCP2 and UCP3 gene clusters could play important roles in energy metabolisms and body mass regulation, and polymorphisms in these two genes might contribute to obesity [4]–[7]. A number of polymorphisms have been well-studied in the UCP2 and UCP3 genes, which include three common single nucleotide polymorphisms (SNPs): two in the UCP2, a promoter variant, -866G>A (rs659366), and a missense polymorphism in codon 55 changing an alanine to a valine (Codon 55Ala/Val rs660339); one in the UCP3, a promoter variant, -55 C/T (rs1800849).

However, the impact of UCP2 and UCP3 polymorphisms on obesity susceptibility is still under debate. Contradictory results have been reported. To further examine their potential role in influencing obesity susceptibility, we performed a meta-analysis on eligible case-control studies to estimate their effects in populations of Asian and European descents.

## Methods

### 1 Search strategy

We performed an exhaustive search on studies that examined the association of the UCP2 and UCP3 gene polymorphisms with obesity. Data were collected from the PubMed, Embase, Web of Science and CNKI, CBMdisc databases and completed on September, 2012. We searched the articles using the search terms UCP2; UCP3; uncoupling protein 2; uncoupling protein 3; variant; polymorphism in combination with obesity. Further, we reviewed all abstracts obtained from our search for relevance; manually reviewed bibliographies and review articles for additional citations and obtained the full text of all potentially relevant articles. All searches were conducted independently by two investigators.

### 2 Study selection

#### Inclusion criteria

(1) A case-control study; (2) numbers in case and control groups reported each allele or genotype; (3) sufficient published data to calculate an odds ratio (OR) with 95% confidence interval (CI).

#### Exclusion criteria

(1) review articles; (2) case reports; (3) abstracts and editorials; (4) reports with incomplete data; (5) studies based on pedigree data; (6) genotype distribution of the controls deviated from Hardy – Weinberg equilibrium (HWE).

### 3 Data extraction

Two investigators independently extracted data, discussed disagreements, and reached consensus on all items. The following information was extracted from each study: the first author's name, year of publication, ethnic origin of the studied population, study design, genotyping methods, number of cases and controls, available allele and genotype frequencies information, and OR with 95% CI. Not all the papers reported the necessary statistics directly so we had to transform and estimate odds ratio from the reported data as necessary, and we did not define any minimum number of patients for a study to be included in our meta-analysis.

### 4 Statistical analysis

HWE of the genotype distribution from the controls was tested by a goodness-of-fit χ2 analysis. The distribution was considered deviated from HWE at *P*<0.05. The strength of association between the UCP2 -866G/A, Ala55Val and UCP3 -55C/T polymorphisms and obesity was assessed by calculating OR with 95% CI in the additive, dominant, recessive and co-dominant models, respectively. The significance of the pooled OR was determined by the Z-test, and *P*<0.05 was considered statistically significant, stratified analysis was also performed analysis on ethnicity, study design and genotyping methods, respectively. The heterogeneity between the studies was evaluated with x2-based Q statistic and *I2* metric. Heterogeneity was considered significant at *P*<0.05 for the Q statistic and *I2* >50% for the *I2* metric. The pooled OR was calculated by a fixed effect model (using the Mantel-Haenszel method) or a random effect model (using the DerSimonian-Laird method) according to the heterogeneity among studies [8], [9]. The false-positive report probability (FPRP) test of Wacholder *et al*. [10] was applied to address the issue of false-positive SNP associations. The potential publication bias was estimated using the modified Begg's and Egger's tests. The significance of the intercept was determined by the t-test suggested by Egger's test (*P*<0.05 was considered representative of statistically significant publication bias). All statistical analyses were conducted by using STATA version 11.0 (Stata Corporation, College Station, TX, USA).

## Results

### 1 Study characteristics

A total of 22 published articles [11]–[32] with 32 outcomes met the inclusion criteria, flow diagram of study identification are shown in Figure S1. The allele and genotype distributions in the included studies are summarized in Tables 1, 2, and 3 for the UCP2 -866G/A, UCP2 Ala55Val and UCP3 -55C/T polymorphisms respectively. Twelve studies examined the association between the UCP2 -866G/A polymorphism and obesity risk [11]–[22], nine studies for the UCP2 Ala55Val polymorphism [16], [22]–[29] and eight for the UCP3 -55C/T polymorphism [15]–[17], [28]–[32].

**Table 1:** Characteristics of the UCP2 -866G/A polymorphism allelic and genotype distribution for obesity risk in studies included in the meta-analysis.

Authors [ref.]	Year	Ethnicity	Study design	methods	Total/Genotypes(GG/GA/AA)		G allele frequency (%)		OR(95% CI)
					Cases	Controls	Cases	Controls	
Esterbauer H et al [11]	2001	European	PCC	PCR-RFLP	340(156/140/44)	256(85/127/44)	66.5	58.0	1.44(1.13–1.82)
Dalgaard LT et al [12]	2003	European	PCC	PCR-RFLP	749(292/322/135)	816(299/369/148)	60.5	59.3	1.05(0.91–1.21)
Mancini FP et al [13]	2003	European	PCC	PCR-RFLP	198(96/82/20)	374(183/165/26)	69.2	71.0	0.92(0.70–1.20)
Schauble N et al [14]	2003	European	PCC	PCR-RFLP	277(108/135/34)	188(72/89/27)	63.4	62.0	1.06(0.81–1.39)
Ochoa MC et al [15]	2007	European	HCC	PCR-RFLP	193(79/80/34)	170(59/92/19)	61.7	61.8	1.00(0.74–1.34)
Wang TN et al [16]	2007	Asian	PCC	PCR-RFLP	324(193/115/16)	114(81/28/5)	77.3	83.3	0.68(0.46–1.01)
Kring SI et al [17]	2008	European	PCC	PCR-RFLP	225(88/96/41)	294(114/131/49)	60.4	61.1	0.97(0.76–1.25)
Heidari J et al [18]	2010	Asian	PCC	PCR-RFLP	75(16/48/11)	75(27/41/7)	53.3	63.3	0.66(0.42–1.05)
Srivastava N et al [19]	2010	Asian	HCC	PCR-RFLP	200(73/86/41)	240(106/113/21)	58.0	67.7	0.66(0.50–0.87)
Zhou HY et al [20]	2011	Asian	PCC	PCR-RFLP	590(176/281/133)	2227(623/1115/489)	53.6	53,0	1.03(0.90–1.17)
Andersen G et al [21]	2012	European	PCC	KASPar	1547(583/754/210)	3153(1133/1499/521)	62.1	59.7	1.10(1.01–1.21)
					2455(874/1183/398)	1567(534/799/234)	59.7	59.6	1.01(0.92–1.11)
Oktavianthi S et al [22]	2012	Asian	PCC	PCR-RFLP	142(38/77/27)	136(54/63/19)	53.9	62.9	0.69(0.49–0.97)
					75(23/42/10)	250(72/133/45)	58.7	55.4	1.14(0.79–1.65)

HCC, hospital-based case-control study; PCC, population-based case-control study; PCR-RFLP, Polymerase Chain Reaction – Restriction Fragment Length Polymorphism.

**Table 2:** Characteristics of the UCP2 Ala55Val polymorphism allelic and genotype distribution for obesity risk in studies included in the meta-analysis.

Authors[ref.]	Year	Ethnicity	Study design	methods	Total/Genotypes(CC/CT/TT)		C allele frequency (%)		OR(95% CI)
					Cases	Controls	Cases	Controls	
Urhammer SA etal [23]	1997	European	PCC	PCR-RFLP	144(41/67/36)	182(56/86/40)	50.7	54.4	0.90(0.66–1.23)
Kubota T et al [24]	1998	Asian	PCC	PCR-RFLP	42(15/13/14)	218(64/97/57)	51.2	51.6	0.98(0.62–1.57)
Otabe S et al [25]	1998	European	HCC	PCR-RFLP	72(ND)	120(ND)	72.2	62.3	1.56(1.00–2.44)
Xiu LL et al [26]	2004	Asian	HCC	PCR-RFLP	119(43/50/26)	177(82/81/14)	57.1	69.2	0.59(0.42–0.83)
Yang M et al [27]	2004	Asian	HCC	PCR-RFLP	99(36/45/18)	57(19/29/9)	59.1	58.8	1.01(0.63–1.62)
Wang TN et al [16]	2007	Asian	PCC	PCR-RFLP	324(211/104/9)	114(90/24/0)	81.2	89.5	0.51(0.32–0.81)
Kosuge K et al [28]	2008	Asian	HCC	Taqman	182(56/80/46)	369(91/199/79)	52.7	51.6	1.05(0.81–1.34)
Mottagui-TabarS et al [29]	2008	European	PCC	DASH	281(57/136/88)	444(90/227/127)	44.5	45.8	0.95(0.77–1.17)
Oktavianthi S et al [22]	2012	Asian	PCC	PCR-RFLP	142(54/77/11)	136(72/49/15)	65.1	71.0	0.76(0.53–1.09)
					75(31/35/9)	250(98/107/45)	64.7	60.6	1.19(0.81–1.74)

ND, no data; DASH, dynamic allele-specific hybridization.

**Table 3:** Characteristics of the UCP3 -55C/T polymorphism allelic and genotype distribution for obesity risk in studies included in the meta-analysis.

Authors[ref.]	Year	Ethnicity	Study design	methods	Total/Genotypes(CC/CT/TT)		C allele frequency (%)		OR(95% CI)
					Cases	Controls	Cases	Controls	
Schrauwen P et al [30]	1999	Asian	PCC	PCR-RFLP	37(24/10/3)	30(23/7/0)	78.4	88.3	0.48(0.18-1.25)
Dalgaard LT et al [31]	2001	European	PCC	PCR-RFLP	791(ND)	915(ND)	74.0	73.1	1.04(0.90-1.22)
Alonso A et al [32]	2005	European	HCC	PCR-RFLP	157(101/50/6)	150(85/61/4)	80.3	77.0	1.21(0.82-1.79)
Ochoa MC et al [15]	2007	European	HCC	PCR-RFLP	184(123/55/6)	157(114/41/2)	81.8	85.7	0.75(0.50-1.13)
Wang TN et al [16]	2007	Asian	PCC	PCR-RFLP	324(216/94/14)	114(84/27/3)	81.2	85.5	0.73(0.48-1.11)
Kring SI et al [17]	2008	European	PCC	PCR-RFLP	220(129/78/13)	310(159/132/19)	76.4	72.6	1.22(0.92-1.62)
Kosuge K et al [28]	2008	Asian	HCC	Taqman	181(88/80/13)	369(172/161/36)	70.7	68.4	1.11(0.85-1.47)
Mottagui-TabarS et al [29]	2008	European	PCC	DASH	286(33/124/129)	469(40/199/230)	33.2	29.7	1.17(0.94-1.47)

### 2 Meta-analysis

**Figure 1:**
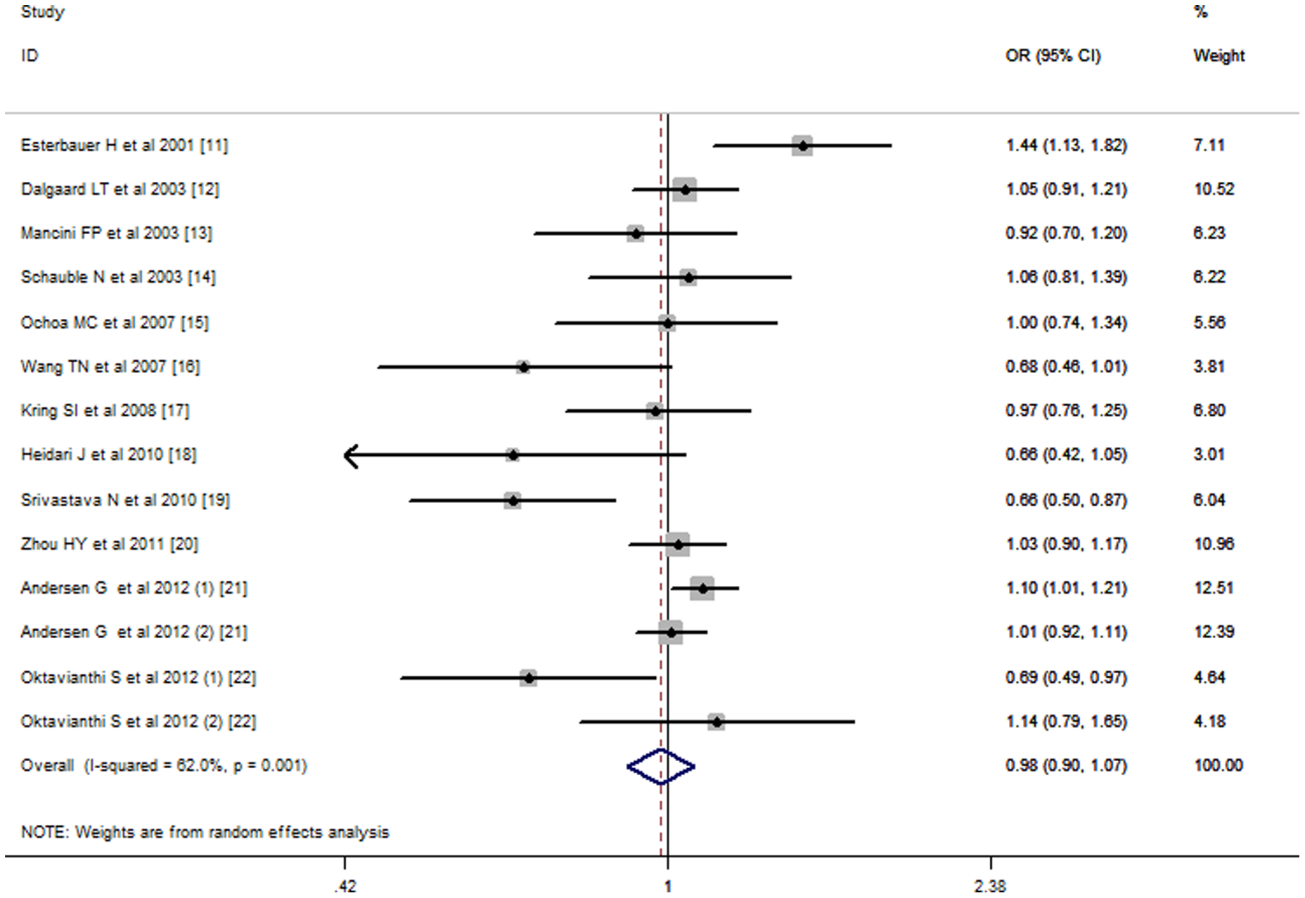
Stratified analysis pooled odds ratios for the association between the UCP2-866G/A polymorphism and susceptibility to obesity. The area of the squares reflects the study-specific weight. The diamond shows the summary random-effects odds ratio estimate from 12 studies.

The association between the UCP2 -866G/A polymorphism and obesity was investigated in 12 studies with a total of 7,390 cases and 9,860 controls. No significant association between the UCP2 -866G/A polymorphism and the risk of obesity was found using either additive (G allele vs. A allele, REM OR = 0.98, 95% CI: 0.90–1.07), dominant (GG/GA vs. AA, REM OR = 0.94, 95% CI: 0.80-1.10), recessive (GG vs. GA/AA, REM OR = 1.01, 95% CI: 0.90–1.14), or co-dominant (GG vs. AA, REM OR = 0.94, 95% CI: 0.78–1.14) models (Table 4 and Fig. 1). However, after stratified by ethnicity, a significant association was revealed in an additive model in populations of European descent (FEM OR = 1.06, 95% CI 1.01–1.12), but not Asian descent (REM OR = 0.81, 95% CI 0.65–1.01) (Table 4). Furthermore, the FPRP value for the UCP2 -866G/A polymorphism in Europeans suggested almost 20% chance of the result being a false positive when assigned a relatively low probability range (i.e. 0.1–0.25) (data not shown), suggesting that the FPRP value is not robust. And only the association in the recessive model (FEM OR = 1.11, 95% CI 1.02–1.18) was in accordant with the result in the additive model, not the dominant (REM OR = 1.01, 95% CI 0.85–1.20) or co-dominant model (REM OR = 1.08, 95% CI 0.91–1.29). These results warrant confirmation by further studies. In addition, after stratified by study design or genotyping methods, no significant association was observed for UCP2 -866G/A polymorphism in both PCC (REM OR = 1.05, 95% CI 0.98–1.12) and HCC (REM OR = 0.84, 95% CI 0.57–1.26), and the same in both PCR-RFLP and others (Table S1).

**Table 4:** Pooled measures for the association between the UCP2–866G/A, Ala55Val and UCP3–55C/T polymorphisms and susceptibility to obesity.

SNPs	Inherited model	Ethnicity	Studies(cases/controls)	I2(%)	FEM		REM	
					OR(95%CI)	P	OR(95%CI)	P
−866G/A	Additive (G vs. A)	overall	12(7390/9860)	62.0	1.02(0.98–1.07)	0.340	0.98(0.90–1.07)	0.097
		Asian	5(1406/3042)	69.7	0.90(0.81–1.00)	0.040	0.81(0.65–1.01)	0.057
		European	7(5984/6818)	28.0	1.06(1.01–1.12)	0.031	1.06(0.99–1.14)	0.097
	Dominant (GG+GA vs. AA)	overall	12(7390/9860)	55.7	1.00(0.92–1.09)	0.746	0.94(0.80–1.10)	0.455
		Asian	5(1406/3042)	58.6	0.88(0.74–1.05)	0.165	0.81(0.56–1.17)	0.252
		European	7(5984/6818)	52.5	1.04(0.94–1,15)	0.433	1.01(0.85–1.20)	0.916
	Recessive (GG vs. GA + AA)	overall	12(7390/9860)	55.8	1.05(0.99–1.12)	0.128	1.01(0.90–1.14)	0.845
		Asian	5(1406/3042)	65.3	0.88(0.76–1.02)	0.098	0.76(0.56–1.02)	0.067
		European	7(5984/6818)	14.9	1.11(1.02–1.18)	0.012	1.11(1.01–1.21)	0.022
	Co-dominant (GG vs. AA)	overall	12(7390/9860)	61.4	1.04(0.94–1.14)	0.455	0.94(0.78–1.14)	0.552
		Asian	5(1406/3042)	68.9	0.84(0.69–1.04)	0.108	0.67(0.41–1.10)	0.115
		European	7(5984/6818)	44.7	1.10(0.99–1.23)	0.089	1.08(0.91–1.29)	0.365
Ala55Val	Additive (C vs. T)	overall	9(1483/2067)	59.7	0.92(0.83–1.02)	0.105	0.91(0.76–1.08)	0.276
		Asian	6(983/1321)	62.4	0.86(0.75–0.99)	0.03	0.84(0.67–1.06)	0.145
		European	3(497/746)	55.5	1.00(0.85–1.17)	0.994	1.04(0.80–1.36)	0.755
	Dominant (CC+CT vs. TT)	overall	8(1411/1947)	42.8	0.83(0.69–1.10)	0.057	0.82(0.62–1.09)	0.169
	Recessive (CC vs. CT+TT)	overall	8(1411/1947)	54.8	0.89(0.76–1.05)	0.166	0.89(0.69–1.13)	0.328
	Co-dominant (CC vs. TT)	overall	8(1411/1947)	40.8	0.81(0.64–1.02)	0.07	0.85(0.62–1.16)	0.304
−55C/T	Additive (C vs. T)	overall	8(2180/2514)	34.4	1.05(0.96–1.16)	0.26	1.04(0.91–1.19)	0.54
		Asian	3(542/513)	57.1	0.94(0.75–1.18)	0.57	0.94(0.55–1.28)	0.42
		European	5(1638/2001)	17.4	1.08(0.97–1.20)	0.13	1.08(0.96–1.23)	0.21
	Dominant (CC+CT vs. TT)	overall	7(1389/1599)	41	1.08(0.91–1.28)	0.37	1.06(0.84–1.33)	0.62
	Recessive (CC vs. CT+TT)	overall	7(1389/1599)	0	1.08(0.85–1.36)	0.54	1.09(0.86–1.39)	0.47
	Co-dominant (CC vs. TT)	overall	7(1389/1599)	10	1.12(0.82–1.54)	0.47	1.13(0.79–1.61)	0.52

FEM:fixed effect pooled measure; REM:randomed effect pooled measure.

**Figure 2:**
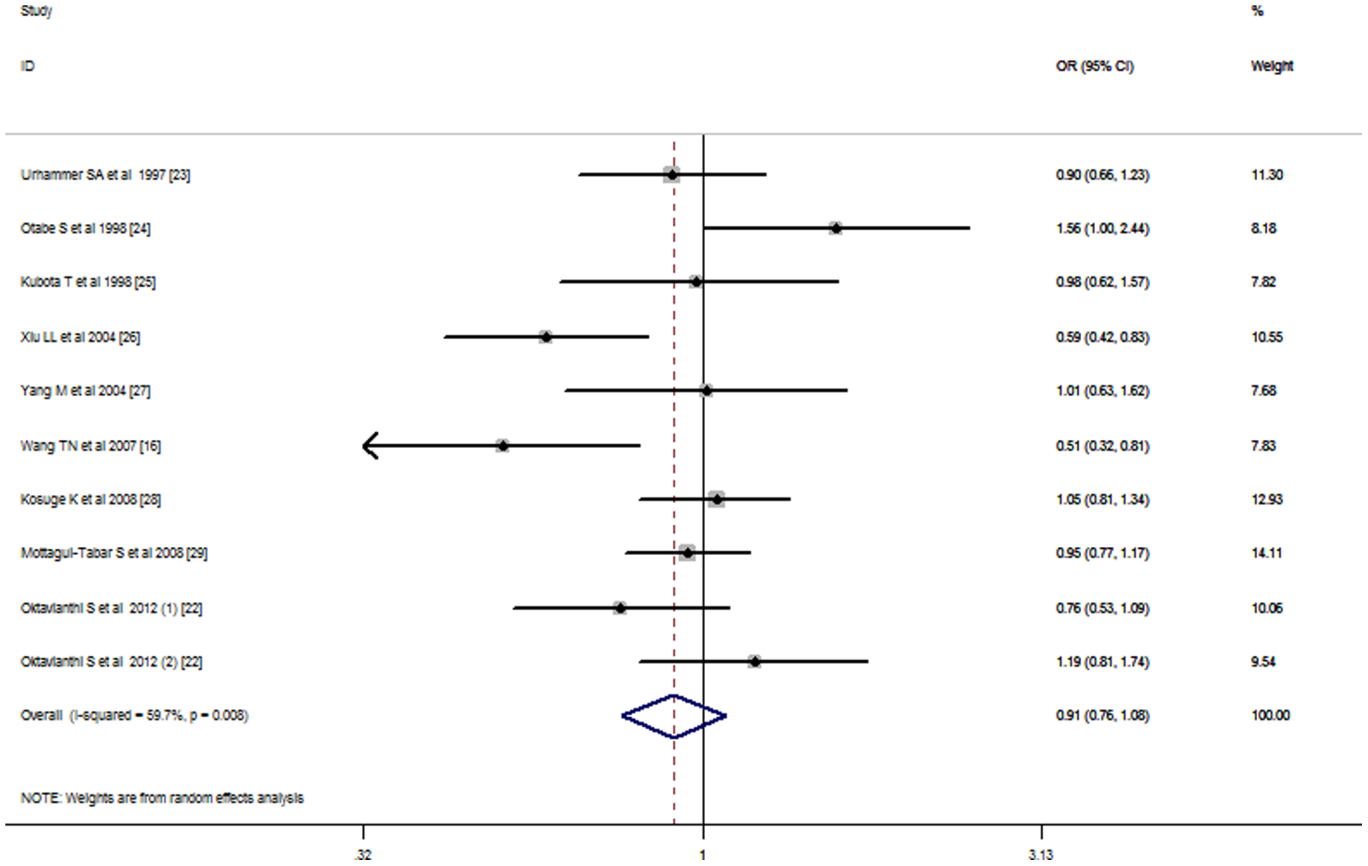
Stratified analysis pooled odds ratios for the association between the UCP2 Ala55Val polymorphism and susceptibility to obesity by ethnicity. The area of the squares reflects the study-specific weight. The diamond shows the summary random-effects odds ratio estimate from 9 studies.

For the UCP2 Ala55Val polymorphism, the C allele was found to be not associated with obesity risk using either additive (REM OR = 0.91, 95% CI: 0.76–1.08), dominant (FEM OR = 0.83, 95% CI: 0.69–1.10), recessive (REM OR = 0.89, 95% CI: 0.69–1.13) or co-dominant (FEM OR = 0.81, 95% CI: 0.64–1.02) model (Table 4 and Fig. 2). Further, no significant association was observed in all genetic models after stratified for ethnicity, study design or genotyping methods (Table 4 and Table S1).

**Figure 3:**
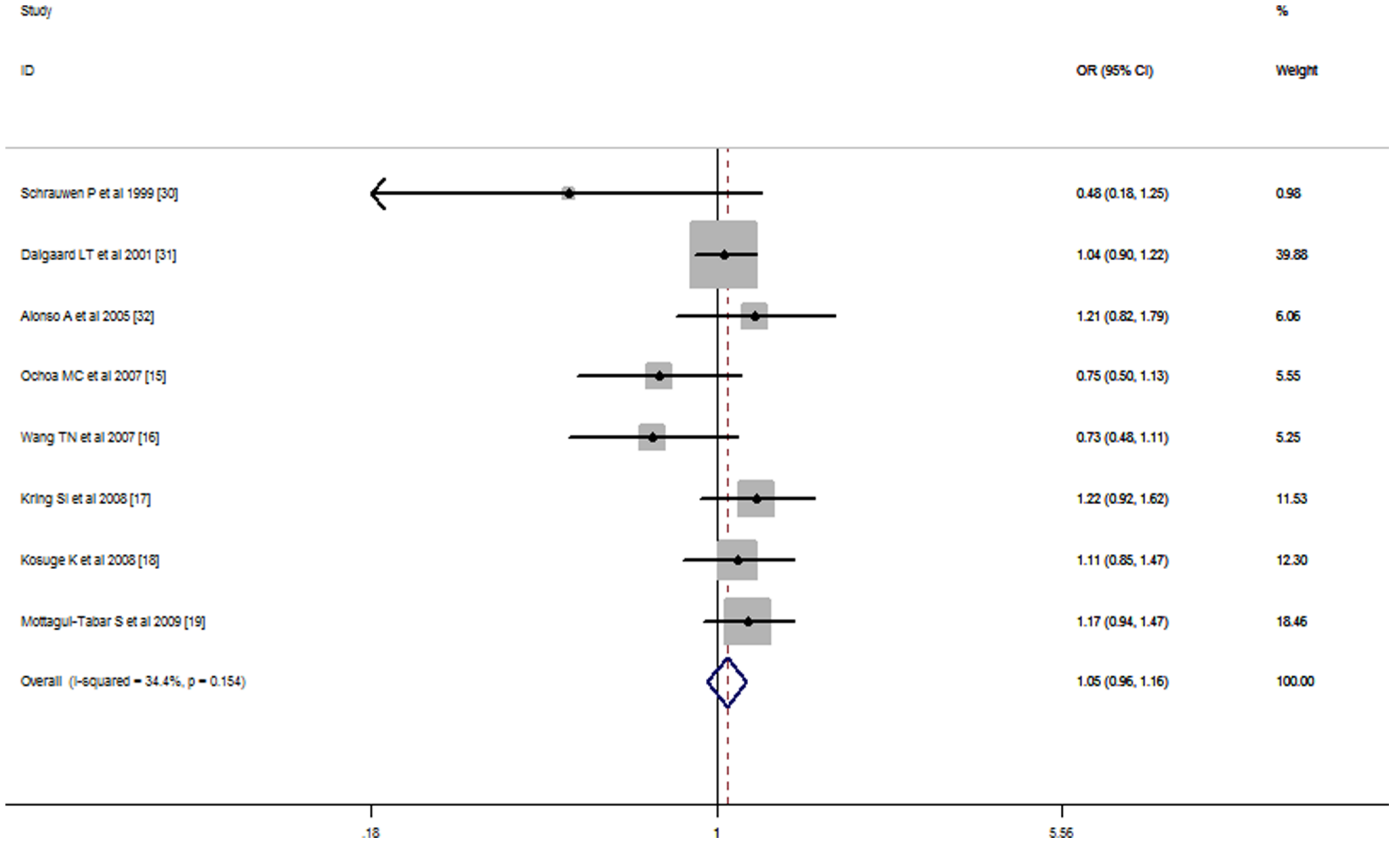
Stratified analysis pooled odds ratios for the association between the UCP3-55C/T polymorphism and susceptibility to obesity by ethnicity. The area of the squares reflects the study-specific weight. The diamond shows the summary fixed-effects odds ratio estimate from 8 studies.

Our meta-analysis also showed no significant association between the UCP3 -55C/T polymorphism and the risk of obesity in all genetic models (additive FEM OR = 1.05, 95% CI: 0.96–1.16; dominant FEM OR = 1.08, 95% CI: 0.91–1.28; recessive FEM OR = 1.08, 95% CI: 0.85–1.36; co-dominant FEM OR = 1.12, 95% CI: 0.82–1.54) (Table 4 and Fig. 3). As the UCP2 Ala55Val polymorphism, no significant association was revealed with the UCP3 -55C/T polymorphism after stratified for ethnicity, study design or genotyping methods in all genetic models (Table 4 and Table S1).

### 3 Heterogeneity and sensitivity analyses

As shown in Table 4, significant heterogeneity was observed among studies of the UCP2 -866G/A, Ala55Val polymorphisms in the overall populations, but no heterogeneity was found in the all models for the UCP3 -55C/T polymorphism in the overall populations. However, when the data were stratified by ethnicity, the heterogeneity between the studies of the UCP2 -866G/A polymorphism was eliminated in populations of European in all genetic models, but not Asian descent. The heterogeneity was also existed in studies of the UCP2 Ala55Val polymorphism both in Asian descent and European descent. To identify the studies with the greatest impact on the overall between-study heterogeneity, sensitivity analyses were conducted in the overall population. Influence of each study on the pooled OR was examined by repeating the meta-analysis with one study excluded at each time. Results (data not shown) show that there is no significant change of the pooled OR, and thus indicates the robustness of our findings.

### 4 Evaluation of publication bias

Funnel plots and Egger's test were performed to assess the publication bias of the literature, as shown in Figure S2 & Table S2. As expected, symmetrical funnel plots were obtained in each of the SNPs tested in all genetic models. And Egger's test further confirmed no publication bias for any of the polymorphisms examined, indicating that our results are statistically reliable.

## Discussion

Obesity is a disorder with a strong genetic component and the impact of hereditary factors is estimated to be between 50% and 85% [33]. Excepting for the robust association between the FTO gene and BMI that was discovered and replicated in genome-wide association studies [34], [35], no specific gene variants explained the common forms of obesity, although mutations in specific genes contributed to a few rare cases of monogenic forms of human obesity. Recently, numerous studies have examined the associations between the three common variants in the UCP2-UCP3 gene cluster and diabetes or obesity risk, including UCP2 -866 G/A, Ala55Val C/T and UCP3 -55 C/T polymorphism. Our previous meta-analyses [36] indicate that the UCP2 Ala55Val and UCP3 -55C/T polymorphisms are type 2 diabetes susceptibility loci in populations of Asian, but not European descent. Meanwhile, the UCP2 -866G/A polymorphism is not a candidate for susceptibility to type 2 diabetes in any ethnic population. However, the impact of UCP2 and UCP3 polymorphisms on obesity is still under debate. Contradictory results have been reported in different populations. To explore the true association between obesity and these three variants, we conducted a meta-analysis of 22 published articles from populations of different ethnic origins.

Here our results indicate that the UCP2-866G/A polymorphisms are obesity susceptibility loci in populations of European, but not Asian population. Ethnic difference between Europeans and Asians provide us a better understanding that structural chromosome variations may affect genes' expression in different ethnic populations and cause ethnic phenotypic diversity or ethnic specific diseases [37]. For the UCP2 -866G/A polymorphism, studies have indicated that in adipocytes, the -866 A-allele was associated with either increased [11] or decreased [38] levels of adipose tissue UCP2 mRNA. The results maybe furnish a basis for understanding the differentiation of -866G/A polymorphism across ethnic groups. Also many of variables that varied between different ethnic origins might be responsible for this phenomenon, including environment, physical activity, lifestyle, *etc*. In addition, the FPRP value for the UCP2 -866G/A polymorphism in Europeans is not robust, which suggest the result warrants confirmation by further studies.

Heterogeneity is potentially a significant problem when interpreting the results of any meta-analysis of genetic association studies [39]. To determine the amount of heterogeneity that existed among these variants, we did an x2-based Q test. Significant between-study heterogeneity in most of the models were used to examine the associations of the UCP2 -866G/A and Ala55Val polymorphisms. Many possible factors, such as ethnicity, the source of the controls, genotyping methods, gender bias, age bias, are responsible for this heterogeneity. After further stratified analysis by ethnicity, study design or genotyping methods, heterogeneity still existed in the association for UCP2 -866G/A and Ala55Val polymorphism. The reason for this is unclear, but might indicate that populations of different ethnicity may also have environmental differences that impact their sensitivity to particular genomic variants. For the heterogeneity existed in most genetic models that we used to analyze the association. We conducted sensitivity analyses by repeating the meta-analysis with one study excluded at each time. The results showed that none of the individual study dramatically influenced the heterogeneity or pooled ORs both in European descent and in Asian descent. Meta-regression or more precise analysis was adjusted by other covariates including age, sex, lifestyles and family history couldn't be finished due to incomplete data. We analyzed the association between these three polymorphisms and obesity by both the random and fixed effects, and the results were consistent in principle.

The results of the present meta-analysis should also be interpreted within the context of its limitations. First, obesity is a complex status involves complex interactions of genes, environment, and behavior. Many studies [40]–[42] revealed that different effect of these three polymorphisms in UCP2 andUCP3 depending on the physical activity and lifestyles. However, it should be kept in mind that BMI is one phenotype of obesity, other phenotypes included fat body mass index, waist circumference, waist for given BMI, intra-abdominal adipose tissue, hip circumference and *etc*. Kring SI *et al* [17] suggested that UCP2 -866G/A was associated with fat body mass index but not BMI. We had insufficient data to take confounder factors such as physical activity, lifestyle, gender and other obesity phenotypes into account in our meta-analysis. Second, some studies [15], [28] suggested that the effect of adjacent loci in the same haplotype should be considered. We again had insufficient data to conduct this. Third, sample size is a limitation of our meta-analysis, especially in the UCP2 -866 G/A and UCP3 -55 C/T polymorphism.

Despite these limitations, our results indicate that the UCP2 -866G/A polymorphism may be obesity susceptibility loci in populations of European, but not Asian population. However, the UCP2 Ala55Val and the UCP3 −55C/T polymorphisms are not candidate loci for susceptibility to obesity in any ethnic population. Maybe this association is not robust and could be due to chance, and additional larger studies that allow stratification for other gene-gene and gene-environment should also be conducted in future analyses.

## Supporting Information

Figure S1 **Systematic review flow diagram.** We performed an exhaustive search on studies that examined the association of the UCP2 and UCP3 gene polymorphisms with obesity. Data were collected from the PubMed, Embase, Web of Science, CBMdisc and CNKI databases and completed on September, 2012. n, number of studies.
(TIF)

Figure S2 **Begg**'**s funnel plot for publication bias test of UCP2 -866 G/A (A), Ala55Val (B) and UCP3 -55 C/T polymorphism (C) and obesity risk.**
(DOC)

Table S1 **Pooled measures for the association between the UCP2 -866G/A, Ala55Val and UCP3 -55C/T polymorphisms and susceptibility to obesity by study design or methods.** HCC, hospital-based case-control study; PCC, population-based case-control study; PCR-RFLP, Polymerase Chain Reaction – Restriction Fragment Length Polymorphism; DASH, dynamic allele-specific hybridization.
(DOC)

Table S2 **Egger**'**s publication bias test for the UCP2 -866G/A, Ala55Val and UCP3 -55C/T polymorphisms in obesity risk.** It was shown that there was no publication bias for any of the polymorphisms examined (*P*<0.05 was considered representative of statistically significant publication bias).
(DOC)
