# Supplementary figures and images for: UCP2 -866G/A, Ala55Val and UCP3 -55C/T Polymorphisms in Association with Obesity Susceptibility — A Meta-Analysis Study
Source: PLoS One. 2013 Apr 1;8(4):e58939. doi: 10.1371/journal.pone.0058939 (PMC3613358; doi:10.1371/journal.pone.0058939)

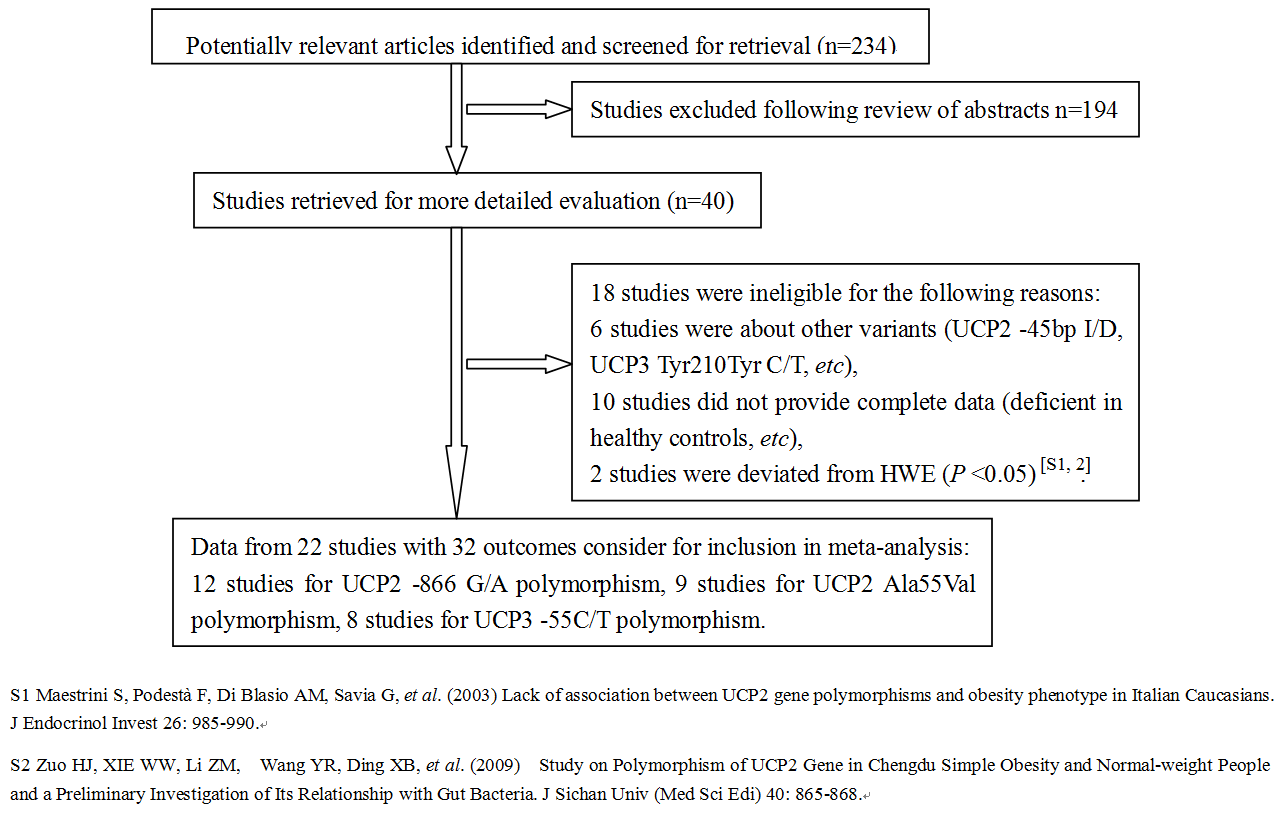

Supplement: Figure S1 — Systematic review flow diagram. We performed an exhaustive search on studies that examined the association of the UCP2 and UCP3 gene polymorphisms with obesity. Data were collected from the PubMed, Embase, Web of Science, CBMdisc and CNKI databases and completed on September, 2012. n, number of studies. (TIF) [file pone.0058939.s001.tif]
