# Supplementary material for: UCP2 -866G/A, Ala55Val and UCP3 -55C/T Polymorphisms in Association with Obesity Susceptibility — A Meta-Analysis Study
Source: PLoS One. 2013 Apr 1;8(4):e58939. doi: 10.1371/journal.pone.0058939 (PMC3613358; doi:10.1371/journal.pone.0058939)
Supplement: Figure S2 — Begg's funnel plot for publication bias test of UCP2 -866 G/A (A), Ala55Val (B) and UCP3 -55 C/T polymorphism (C) and obesity risk. (DOC) [file pone.0058939.s002.doc]

Begg's funnel plot with pseudo 95% confidence limits

log[or]

s.e. of: log[or]

0

.1

.2

.3

-.5

0

.5

**A**

Begg's funnel plot with pseudo 95% confidence limits

log[or]

s.e. of: log[or]

0

.1

.2

.3

-1

-.5

0

.5

**B**

Begg's funnel plot with pseudo 95% confidence limits

log[or]

s.e. of: log[or]

0

.2

.4

.6

-1

-.5

0

.5

1

**C**

Figure S2
