# Supplementary material for: UCP2 -866G/A, Ala55Val and UCP3 -55C/T Polymorphisms in Association with Obesity Susceptibility — A Meta-Analysis Study
Source: PLoS One. 2013 Apr 1;8(4):e58939. doi: 10.1371/journal.pone.0058939 (PMC3613358; doi:10.1371/journal.pone.0058939)
Supplement: Table S1 — Pooled measures for the association between the UCP2 -866G/A, Ala55Val and UCP3 -55C/T polymorphisms and susceptibility to obesity by study design or methods. HCC, hospital-based case-control study; PCC, population-based case-control study; PCR-RFLP, Polymerase Chain Reaction – Restriction Fragment Length Polymorphism; DASH, dynamic allele-specific hybridization. (DOC) [file pone.0058939.s003.doc]

**Table S1**

| **SNPs** | **Stratified analysis** | **Ethnicity** | **Studies** | ***I*^2^ (%)** | **FEM** | | **REM** | |
| --- | --- | --- | --- | --- | --- | --- | --- | --- |
|  |  |  |  |  | **OR(95%CI)** | **P** | **OR(95%CI)** | **P** |
| -866G/A | Study design | PCC | 10 | 43.7 | 1.06(1.01-1.10) | 0.01 | 1.05(0.98-1.12) | 0.174 |
|  |  | HCC | 2 | 55.1 | 0.88(0.69-1.13) | 0.334 | 0.84(0.57-1.26) | 0.406 |
|  | Methods | PCR-RFLP | 12 | 50.1 | 1.05(0.99-1.11) | 0.084 | 1.02(0.97-1.11) | 0.745 |
|  |  | others | 1 | — | — | — | — | — |
| Ala55Val | Study design | PCC | 5 | 44.6 | 0.89(0.78-1.02) | 0.097 | 0.88(0.72-1.06) | 0.170 |
|  |  | HCC | 4 | 76.8 | 0.96(0.81-1.14) | 0.614 | 0.98(0.67-1.43) | 0.914 |
|  | Methods | PCR-RFLP | 7 | 65.8 | 0.87(0.75-0.99) | 0.042 | 0.88(0.69-1.12) | 0.295 |
|  |  | others | 2 | 0 | 0.99(0.84-1.16) | 0.899 | 0.99(0.84-1.16) | 0.899 |
| -55C/T | Study design | PCC | 5 | 45.7 | 1.06(0.95-1.18) | 0.306 | 1.04(0.87-1.24) | 0.688 |
|  |  | HCC | 3 | 38.7 | 1.04(0.85-1.26) | 0.726 | 1.02(0.79-1.32) | 0.868 |
|  | Methods | PCR-RFLP | 6 | 45.7 | 1.02(0.92-1.14) | 0.700 | 0.98(0.82-1.17) | 0.806 |
|  |  | others | 2 | 0 | 1.16(0.95-1.41) | 0.135 | 1.16(0.95-1.41) | 0.135 |
