# Supplementary material for: UCP2 -866G/A, Ala55Val and UCP3 -55C/T Polymorphisms in Association with Obesity Susceptibility — A Meta-Analysis Study
Source: PLoS One. 2013 Apr 1;8(4):e58939. doi: 10.1371/journal.pone.0058939 (PMC3613358; doi:10.1371/journal.pone.0058939)
Supplement: Table S2 — Egger's publication bias test for the UCP2 -866G/A, Ala55Val and UCP3 -55C/T polymorphisms in obesity risk. It was shown that there was no publication bias for any of the polymorphisms examined (P<0.05 was considered representative of statistically significant publication bias). (DOC) [file pone.0058939.s004.doc]

**Table S2**

| **SNPs** | **Genetic models** | | | |
| --- | --- | --- | --- | --- |
|  | **Additive** | **Dominant** | **Recessive** | **Co-dominant** |
| -866G/A | 0.094 | 0.183 | 0.139 | 0.088 |
| Ala55Val | 0.790 | 0.609 | 0.892 | 0.364 |
| -55 C/T | 0.170 | 0.118 | 0.093 | 0.116 |
